# 1188. The Effect Of The COVID-19 Pandemic On Influenza-Related Hospitalization, Intensive Care Admission And Mortality In Canadian Children

**DOI:** 10.1093/ofid/ofab466.1380

**Published:** 2021-12-04

**Authors:** Helen E Groves, Jesse Papenburg, Kayur Mehta, Julie A Bettinger, Manish Sadarangani, Scott Halperin, Shaun Morris

**Affiliations:** 1 The Hospital for Sick Children, Toronto, Toronto, Ontario, Canada; 2 Departments of Pediatrics and Medical Microbiology, McGill University Health Centre, Montreal, QC, Canada, Montreal, Quebec, Canada; 3 McGill University, Montreal, Quebec, Canada; 4 University of British Columbia, Vancouver, British Columbia, Canada; 5 IWK Hlth Ctr, Halifax, NS, Canada; 6 Hospital for Sick Children, University of Toronto, Toronto, Ontario, Canada

## Abstract

**Background:**

The COVID-19 pandemic resulted in unprecedented implementation of wide-ranging public health measures globally. During the pandemic, dramatic decreases in seasonal influenza virus detection have been reported worldwide. Information on pediatric influenza-related hospitalizations is limited. We describe influenza-related hospitalization in Canadian children during the 2020/2021 influenza season compared to ten previous seasons.

**Methods:**

Data on influenza-related hospitalizations, intensive care unit (ICU) admissions and in-hospital deaths in children across Canada were obtained from the Canadian Immunization Monitoring Program, ACTive (IMPACT). This national surveillance initiative comprises 90% of all tertiary care pediatric beds in Canada. The total study period included eleven influenza seasons from September 2010 to April 2021 inclusive. Time series modelling was used to compare trends in influenza-related hospitalizations during the 2020/2021 season (September 2020 to April 2021 inclusive) with the ten previous seasons.

**Results:**

During the 2020/2021 influenza season there were no pediatric influenza infection-related hospitalizations. This was a significant decrease compared to the predicted total influenza-related hospitalizations for this period (p< 0.001). No pediatric ICU admission or deaths were reported for the 2020/2021 influenza season.

**Conclusion:**

We show complete absence of influenza infection-related hospitalization in children in Canada during the 2020/2021 season. This significant decrease is likely related in large part to non-pharmacological public health interventions implemented during the COVID-19 pandemic, although the potential role of viral interference is unknown. Our findings suggest measures such as use of facemasks, hand-washing, distancing and school closures may be beneficial for influenza control and mitigation of future influenza epidemics.

**Disclosures:**

**Helen E. Groves, PhD, MBBCh BAO**, **Abbvie** (Other Financial or Material Support, Dr. Groves reports personal fees from Honoraria received from Abbvie for education meeting presentation, not relevant to the submitted work.) **Jesse Papenburg, MD**, **AbbVie** (Grant/Research Support, Other Financial or Material Support, Personal fees)**Medimmune** (Grant/Research Support)**Sanofi Pasteur** (Grant/Research Support)**Seegene** (Grant/Research Support, Other Financial or Material Support, Personal fees) **Manish Sadarangani, BM BCh, DPhil**, **GlaxoSmithKline** (Grant/Research Support)**Merck** (Grant/Research Support)**Pfizer** (Grant/Research Support)**Sanofi Pasteur** (Grant/Research Support)**Seqirus** (Grant/Research Support)**Symvivo** (Grant/Research Support)**VBI Vaccines** (Research Grant or Support) **Shaun Morris, MD, MPH, DTM&H, FRCPC, FAAP**, **GSK** (Speaker’s Bureau)**Pfizer** (Advisor or Review Panel member)**Pfizer** (Grant/Research Support)

